# En-Face Optical Coherence Tomography Angiography for Longitudinal Monitoring of Retinal Injury

**DOI:** 10.3390/app9132617

**Published:** 2019-06-28

**Authors:** Jonathan Luisi, Wei Liu, Wenbo Zhang, Massoud Motamedi

**Affiliations:** 1 Pharmacology and Toxicology, University of Texas Medical Branch, Galveston, TX 77555, USA; 2 Center for Biomedical Engineering, University of Texas Medical Branch, Galveston, TX 77555, USA; 3 Ophthalmology and Visual Science, University of Texas Medical Branch, Galveston, TX 77555, USA; 4 Neuroscience and Cell Biology, University of Texas Medical Branch, Galveston, TX 77555, USA

**Keywords:** optical coherence tomography, optical coherence angiography, ophthalmic imaging, laser injury, choroidal neovascularization

## Abstract

A customized Optical Coherence Tomography Angiography (OCTA) algorithm and Orthogonal OCT (en-face and B-Scans) were used for longitudinal assessment of retina murine vascular and tissue remodeling comparing photoreceptor ablation and laser-induced Choroidal Neovascularization (CNV). In the mouse model, we utilized a combined OCTA/OCT technique to image and quantify morphological and vascular features of laser lesions over time. This approach enabled us to monitor and correlate the dynamics of retina vascular and tissue remodeling as evidenced by swelling, edema, and scarring. From the OCT B-Scans, three stages of inflammatory progression were identified: the early response occurring within hours to day 3, the transition phase from 3–7 days, and the late stage of 7–21 days entering either the resolving phase or chronic phase, respectively. For the case of CNV, en-face OCTA revealed a transient non-perfusion of inner retina capillaries, specifically Deep Vascular Plexus (DVP), which corresponded to growth in lesions of a height of 200 μm or greater. Non-perfusion first occurred at 24 hours, persisted during edema and CNV formation days 7–14. In contrast, the acute inflammation induced photoreceptor damage, but no detectable alterations to the microvasculature were observed. We demonstrated that the en-face OCTA system is capable of visualizing capillary networks (~5 μm) and the corresponding tissue remodeling and growth dynamics allowing for separating acute injury from CNV. For the first time, by using OCTA we observed the presence of the 5–10 μm capillary non-perfusion present in DVP as part of CNV formation and the associated wound healing in the retina.

## 1. Introduction

Recent advances in the fields of cellular and molecular biology have determined that the biological and pathophysiological processes involved in the onset and progression of retina neuroinflammatory and degenerative diseases are inflammation accompanied by tissue and vascular remodeling. An important aspect related to the understanding of retina neurodegeneration that can be studied in animal models is making a distinction between how an injury transitions from acute to either a resolving phase or a chronic response [1–3]. This response is defined as the pro-inflammatory acute or early phase that lasts only a few days before transitioning to the resolving phase where processes such as wound repair and scarring occur. In contrast, chronic inflammation is where the resolving phase does not occur, and instead the pro-inflammatory progression of the acute phase persists long after the initial insult is removed. Specific changes in the different capillary beds in response to injury have not previously been studied in-vivo comparing vascular alterations in acute and chronic phases.

CNV is clinically diagnosed with a variety of image-based methods, but most commonly with fluorescent angiography (FA) using either fluorescein or indocyanine green (ICG) dyes delivered intravascularly as a contrast agent [4,5]. Vascular leakage visualized by ICG or FA indicates the presence of altered vascular permeability; although, rare cases of anaphylaxis may occur with Fluorescein or ICG injections [6]. While FA has utility to be used in the clinic as the procedure is established, the correlation and interpretation of OCTA are not yet fully understood; however, OCTA can show alterations in individual vascular beds.

The murine laser-induced model of CNV (L-CNV) induces an inflammatory response in the Retinal Pigment Epithelial Layer (RPE) that is known to be neurodegenerative and promotes pathological neovascularization [7]. Low-dose laser photocoagulation that is below the CNV threshold can cause photoreceptor ablation, an acute injury. In the acute injury dose range, there are two key thresholds; first is the safety threshold (subvisible [8]) in which photoreceptors are stressed, but there is no immediately observable lesion, and secondly, when the intensity is sufficient to ablate both the photoreceptors and the RPE cells inducing CNV. Currently, the CNV model is used in preclinical treatment efficacy studies, including anti-VEGF [9] and anti-TNFα [10] therapies, where FA and histology are the current gold standards to measure the impact of drug treatment on limiting the severity of CNV. It is important to note that the low dose laser photocoagulation and photoablation, such as used in surgery, results in acute inflammation that may affect the contralateral eye [11], but does not develop into chronic inflammation [12]. Acute injury lesions lead to permanent photoreceptor ablation and subsequent scarring without directly affecting the vasculature [13,14]. Accurate data models that can differentiate between acute and chronic phases are needed to be able to predict the effect of laser injury modulating inflammation [15,16], as the subsequent retinal thickness changes may occur days or weeks later [17,18].

OCT provides the non-invasive ability to create high-resolution depth profiles of the retinal structure without any contrast agent. OCT B-scan imaging is comparable in the resolving power to measure retinal layers via traditional histology [19,20]. In mouse models, the histological findings are well correlated to measurements with the OCT B-scans [21,22]. The C-scan, or en-face OCT scan [23], is a slice through the volume providing a more traditional *fundus-like* view. The C-scans can be combined via projection methods as an OCT *slab*, or a portion of the depth volume, containing capillaries of the relative retinal layer. Clinical OCTA systems have been used to characterize Diabetic Retinopathy (DR) and CNV in human patients after diagnosis by traditional methods [24–26]. Recently, OCTA was used to monitor capillary response and choroid vasculature [27] in laser photocoagulation therapy, which is used to modulate the oxidative demand in proliferative diabetic retinopathy [28,29] and diabetic macular edema (DME). The photoreceptor damage of modern photocoagulation is at the limit of detection using conventional fundus imaging, and OCT only detects macular thinning after several weeks [17]. FA and OCTA have been well correlated in the inner retina when OCTA was used in a diabetic mouse model [30]. Few trials were performed with OCTA in L-CNV [31–33], but longitudinal studies were limited to only the chronic phase’s impact on the choroid.

To better understand the difference in inflammatory response between the acute injury and CNV, we used two distinct laser models. In these procedures, a surgical photoablation laser operating at 532nm was used. The classical L-CNV model is used here to study the chronic phase of the inflammatory response [34]. The acute injury model photoreceptor ablation induces a predominantly acute inflammatory response [35]. Histological analysis of lesions precludes monitoring the dynamics of lesion formation in longitudinal studies; therefore, the method we developed uses high-resolution data cubes that were processed for multiple co-registered analysis techniques orthogonal (cross sections and en-face views) [36] and OCTA. Simply put, one single scan replicates the information of multimodal scanning, providing depth-resolved angiography and perfect co-registration between views. While previous CNV studies focused solely on visualization of the choriocapillaris [32,37], in the current study we are screening for imaging markers of the phases of retinal injury. Therefore, we build upon previous work to show that high-resolution orthagonal OCT and OCTA are a multimodal combination that is highly suitable for longitudinal retinal imaging studies.

## 2. Materials and Methods

### Animal Use:

All animal handling and care protocols were approved by the University of Texas Medical Branch IACUC board, and procedures were performed per the standards set forth in the ARVO Statement for the Use of Animals in Ophthalmic and Visual Research. For retinal imaging, wild-type C57BL/6 mice (The Jackson Laboratory) were used. Both male and female mice were randomly assigned to acute injury and L-CNV groups. Since CNV is a degenerative disorder affecting elderly patients, aged mice (80 weeks) were used in the initial experimental group as they exhibit a realistically variable response, including an increase in RPE autofluorescence, and the aged female BL6 mice develop a severe response to L-CNV [34,38,39]. With aging, the ability for wound healing or regulation of inflammatory response may be impaired [40]. For each of the experimental repetitions, adult mice (18 weeks) were used to verify the findings of the longitudinal group. The experimental groups were chosen as litters of 4-female and 4-male mice. In total, 24 animals were used for in-vivo imaging and confocal imaging of tissue. From the data, complete sets of longitudinal data (16 lesions per condition) were measured at all seven time-points. The aged matched group subjects were screened for any apparent retinal disruptions or defects. Mice were anesthetized with a ketamine dexmedetomidine mixture, and their eyes were dilated with phenylephrine and tropicamide (Alcon). Corneal hydration was maintained with lubricating drops (Alcon).

Laser lesions were induced with an image-guided laser system (Micron III Phoenix Research Laboratories, Pleasanton, CA), to guide a photocoagulation laser (Meridian Merilas) to the precise location on the retina. The focused 532 nm laser beam onto the retina reliably induced 4 lesions in the right eye of each subject, typically at the 3,6,9, and 12 o’clock positions corresponding to the retinal quadrants, as previously described by Gong et. al. [34]. Prior to this experiment, a dosing study was used to determine threshold parameters for reliably inducing acute injury lesions and L-CNV, using the Micron III system in place of a slit lamp ophthalmoscope [35,41]. The output power was verified using a thermopile power meter (Field Max II, Coherent). The L-CNV model [38,42,43] was induced with 180 mW for 70 ms (0.012 joules), and acute injury with 46 mW of power for 200 ms (0.009 joules), with a fixed spot size of 50 μm. Following the laser treatment, we imaged the lesions at the timepoints of 1h, 1 day, 2 days, 1 week, 2 weeks, and 3 weeks.

### In-Vivo Lesion Verification:

**Figure A1.**
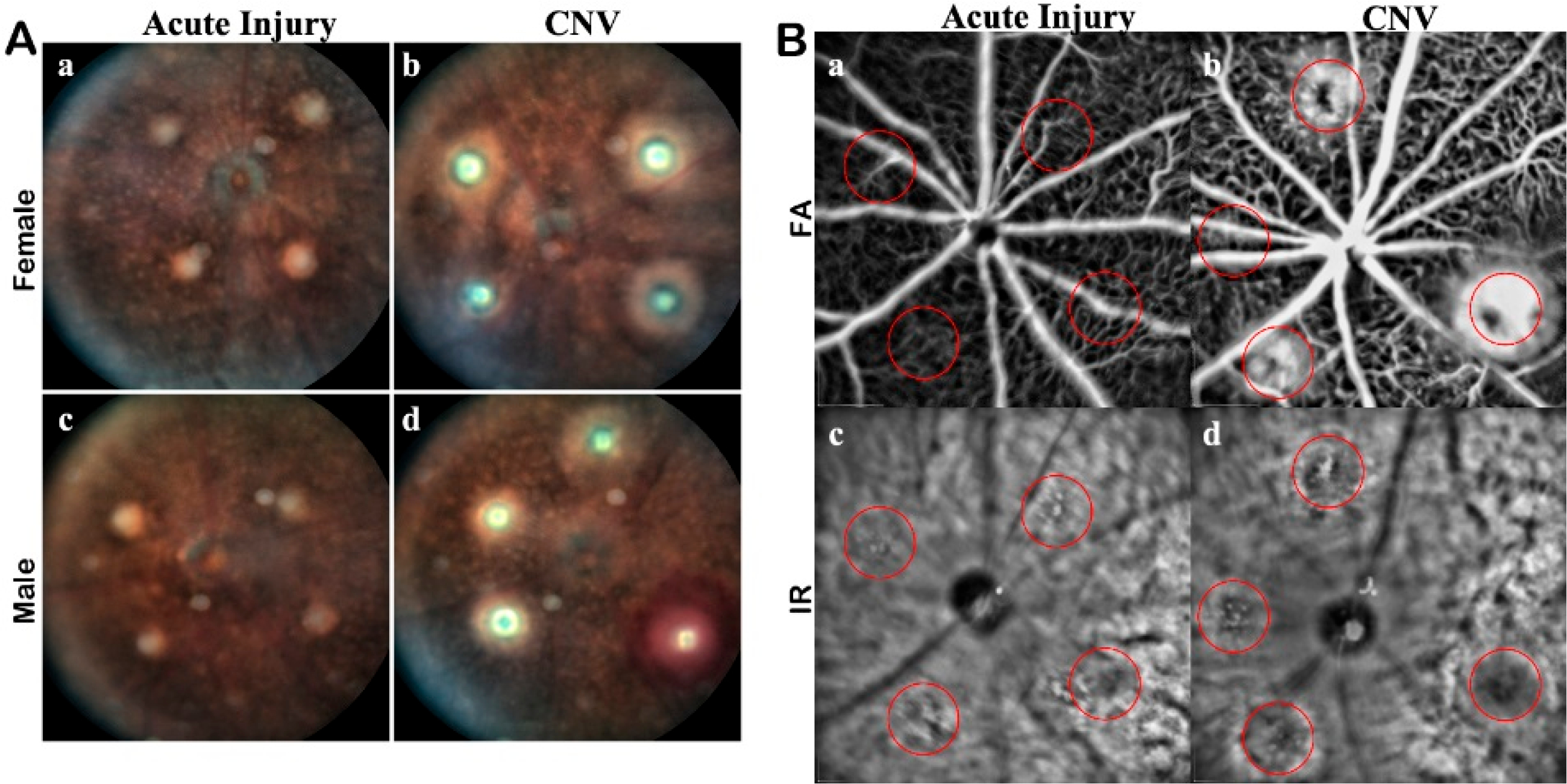
In verifying the effectiveness and consistency of laser lesioning, the fundus imaging of the retinas were reviewed. (**A**): Sample review of the final color fundus image in the Micron III during laser photocoagulation procedure. The sample shows an example of both gender (rows) and treatment groups (columns). The one lesion that exhibited bleeding can be seen in the male CNV example (**d** in (**A**)). (**B**): Sample review of the FA (**a**,**b** in (**B**)) and IR (**c**,**d** in (**B**)) imaging at day 7, corresponding to the male mice in (**A**). In this sample, the absence of leakage in the acute injury is clearly contrasted by the CNV leakage. In the FA CNV images (**b** in (**B**)), the lesion at the 9 o’clock position is a sample of the three lesions that were negative for leakage. The acute injury lesions (bottom left **c**) became hard to discern by day 7 in the IR images, and all FA images (**a** in (**B**)) were negative or inconclusive for leakage.

Each lesion was verified as acute injury or CNV using traditional in-vivo ophthalmology modalities using a Spectralis HRA +OCT imaging system. White light, Infrared (IR), and FA fundus imaging modes were all used to image the retina (Figure A1). In conjunction with the fundus imaging, volumetric scans were acquired with the Bioptigen OCT. Prescreening of each subject was used to establish baseline health, specifically scanning for preexisting retinal degeneration or physiological abnormalities. Any signs of bleeding or redness were noted, as they could be symptomatic of choroidal bleeding or retinal detachment.

As an adjunct to color fundus imaging, IR SLO imaging was used to confirm lesions. For FA imaging, 100 mg/kg of 10% Sodium Fluorescein (AK-FLUOR, Akorn, Decatur, IL) was diluted 1:20 in normal saline for intraperitoneal injection prior to fluorescence fundus imaging with the Spectralis HRA. Multiple FA images were captured through the late stage of perfusion. FA imaging was used to validate vascular leakage from day 3 to 21. Reference IR images were acquired concurrently for comparisons to ensure the focus and accuracy of the FA images. Images were calibrated, and pertinent measurements are in μm or μm^2^. Acute injury lesions were verified as transient hyperreflectivity and the low autofluorescent signal on fundus imaging. The lesions were then verified with FA to show they were not leaking and OCT for assessment of layer involvement.

### En-face OCTA Procedure:

Implementation of OCTA methods were optimized to either extract functional data, such as blood flow, or widefield high-resolution structural data in-vivo [5]. In this study, for wide field analysis, we were prioritizing analyzing structural data over flow data, as the accuracy of flow rate is currently undetermined. To develop OCTA algorithms for resolving the capillary beds, the nature of the imaging system must be considered. An individual OCT depth profile is the A-scan, produced by the interference pattern of the sampling beam to a reference beam. The image is adjustable to compensate for the curvature of the eye by changing the reference arm length in proportion to the axial distance. The B-scan is a cross-section of the retinal volume produced by shifting consecutive A-scan in a line. The B-Scan pattern can be linear or radial, but for our purposes, we use rectangular volumes of consecutive B-scans.

**Figure A2.**
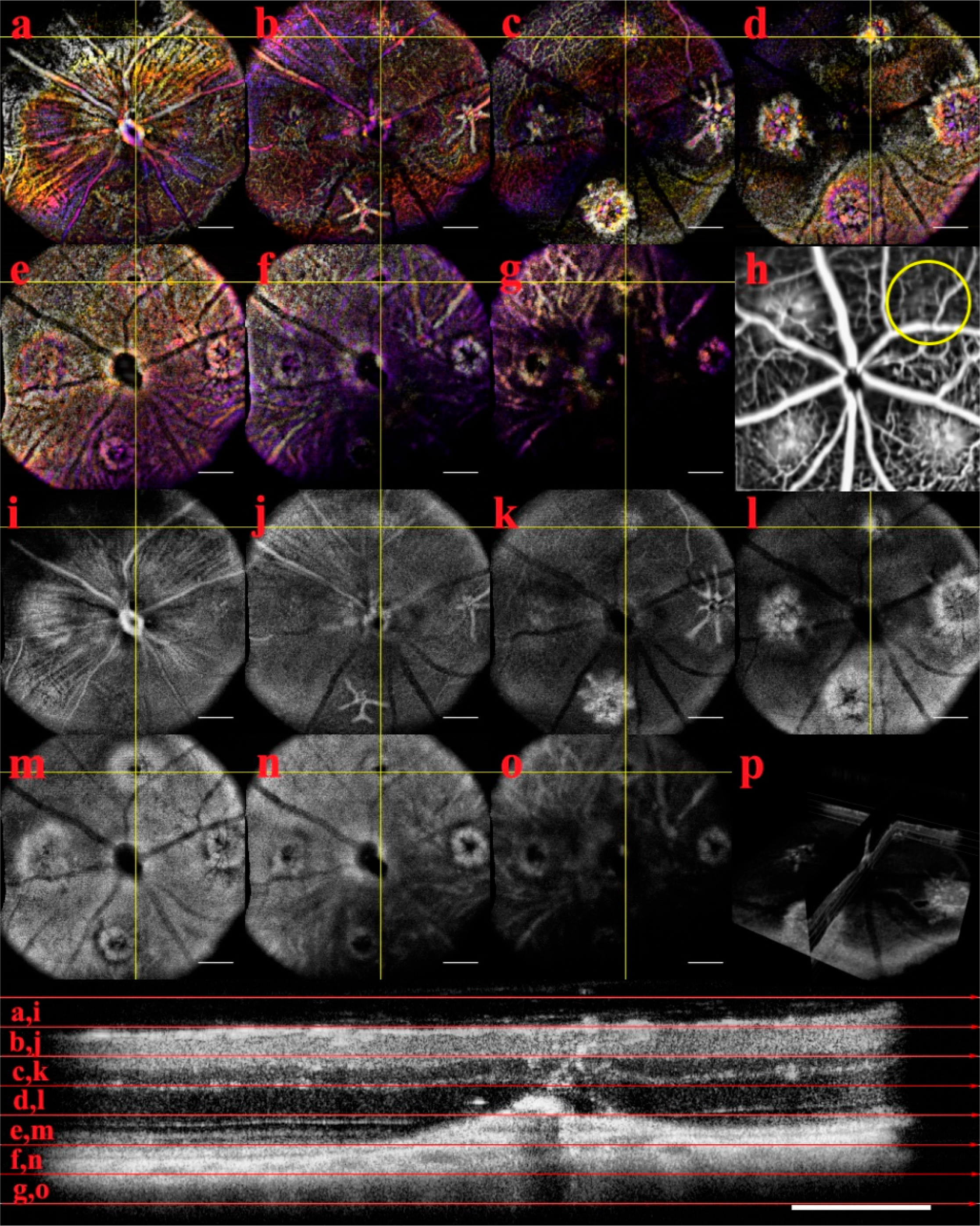
The OCT volume was sequentially re-sliced for en-face views with 60 μm thick optical slabs. The OCTA projections (**a**–**g**) track the lesion marked with yellow crosshairs and circled in the Fluorescence Angiography (**h**). The same optical slabs are presented as traditional en-face views (**i**–**o**) however the edge gradients of the lesions are not well defined, and the boundaries are difficult to determine. (**p**) is the three-dimensional visualization of the orthographic views cutting through the center of the lesion. The bottom B-scan is the horizontal slice through the center of the lesion marked. The Superficial Vascular Plexus is highlighted in A, The Intermediate Vascular Plexus in (**b**), and the Deep Vascular Plexus in C. The Retinal pigment epithelial layer is ruptured as evidenced by the altered structure (**e**–**f**).

To extract OCTA data from the intensity image, a high-density scan is required. For smooth transitions, the C-scan needs to be isotropic in the lateral plane, i.e. the same number of A-scans per both axes in the C-scan. En-face OCTA can be achieved by using a scan density of 1000 × 1000 A-scans, with a lateral resolution of 1.4 μm, as a single volume. The axial resolution of the system is 1.9 μm. Averaging or multiple consecutive scans decreases the area to a smaller region of interest, while samples of the lesions were acquired in this manner, a widefield scan was used for analysis. In the en-face scan, the blood flow in the vasculature increases both scatter (phase) and intensity and has been proposed for use in multi-functional OCT imaging to extract angiography and high-resolution structural information from a single scan volume [44]. With the single high-density volume, post-processing produced multiple outputs including high-resolution B-scans and en-face OCTA (Figure A2). Our custom automated image processing routine was developed in ImageJ to handle the raw data Bioptigen OCT reader and generate the registered 3D data cube that compensated for motion artifacts and misalignment of the B-scans.

From the data cube, high-resolution B-scans were resliced through the center of lesions for analysis of lesion morphology. The measurements recorded in μm were self-normalized for each lesion and reported as percent change. The percent change in the dynamics took the first measurements at one hour and normalized the subsequent measurements as expansion (positive values) or reduction (negative values). Additionally, OCTA was generated by further processing with a custom algorithm on the entire data cube or a VOI (Volume of interest, a 3D region of interest). Our algorithm uses structural enhancement and segmentation of the en-face intensity images to produce the 3D angiography. This method is a distinct approach to the implementation of the theory behind the J. Wang, et al method of reflectance-based projection-resolved (rbPR) OCTA algorithm to reduce shadow artifacts [45]. The rbPR algorithm is a probabilistic approach to histogram equalization and segmentation, whereas our approach is a sequential set of localized structural enhancement iterators. Both approaches enhance the capillary network and are robust to rejecting shadow artifacts in the projections of the retinal layers.

### Lesion Dynamic Descriptors:

**Figure 1.**
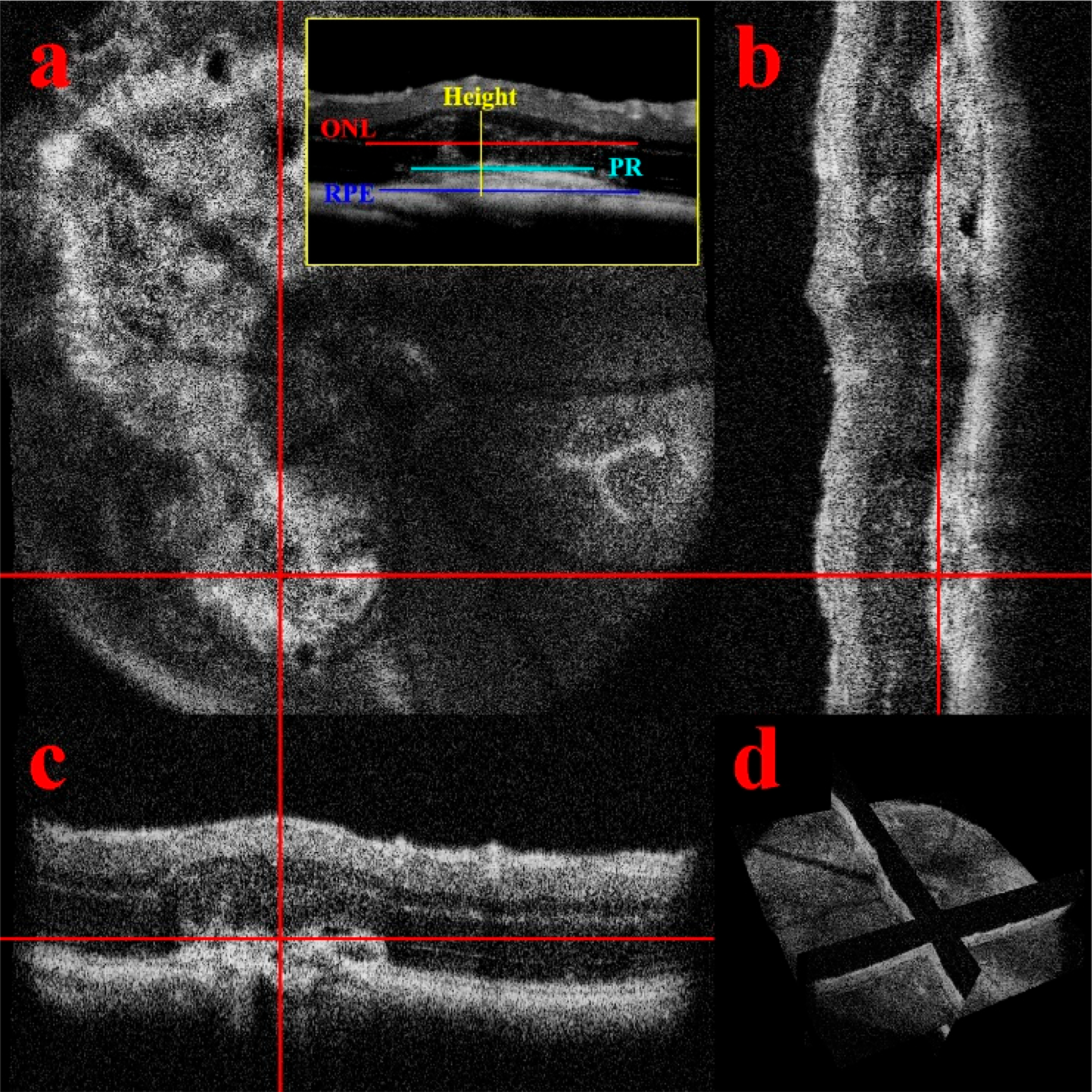
Orthographic views and lesion measurement. The en-face view (**a**) is aligned with two B-scans (**b**,**c**) to determine the center of the lesion. The red registration lines show corresponding coordinates. (**d**) is the three-dimensional view of the projections illustrating that the linked views are digital slices of the volume from the same scan. The inset is the measurement scheme for the key layers of the lesion.

The longitudinal imaging with the OCT system produced data cube (Figure A2) for each timepoint that was processed for en-face images and B-Scans. The qualitative description of the lesion dynamics defined the critical measurements used to differentiate the influence of the light dose on the degree of retinal injury and identify the extent of retinal layers that were disrupted. The lesions were numbered, and B-Scans that crossed through the lesion midpoint were identified for measurements, exemplars in (Figure 1).

### Lesion Dynamics; B-Scan Measurements:

Lesion growth measurements were compiled as an individual lesion by treatment group and gender. Measurements were recorded to capture the lesion growth dynamics; dimensions from the layers were a lateral line across the boundary of the outer nuclear layer and outer plexiform layer (Label: ONL). A line was drawn laterally across the Retinal Pigment Epithelium (Label: RPE) layer from edge to edge of visible disruptions. The height of the lesion (Label: Height) was measured as the maximum point of OPL-RPE disruption. The final measurement was the widest point across the Exterior Limiting Membrane to Inner Segment/Outer Segment (ELM-IS/OS) protrusion into the ONL (Label: PR).

### Retinal and Choroid Flatmounts:

As traditional validation of CNV injury, choroidal flatmounts were prepared for both doses. The corresponding inner retinal was also extracted for flatmounts to show the vascular morphology. Retinas were prepared for confocal imaging by perfusion and/or direct staining with the appropriate fluorescent label. The vasculature was perfusion labeled with Fluorescein-labeled Concanavalin A (Con A) through the carotid artery [46]. All eyes were fixed in 4% PFA and micro-dissected for both retinal and choroid flatmounts. Flatmounts, that were not perfused were incubated with 594 AlexaFluor-labeled Isolectin B4 for 2 hours 1:500 dilution. All samples were stained with 1:200 DAPI solution for 30 minutes. The flatmounts were imaged on the confocal microscope to validate the CNV model.

### Statistics:

For the in-vivo visualization with OCTA, there were 8 mice of mixed sexes and two groups of old and young for each condition. To prevent errors from variable group size effects, a constant 16 lesions that were verified imaged at all time points were used. For immunofluorescent staining, there were an additional 16 mice used to verify the in-vivo findings. The standard deviation σ and average μ were computed for individual groups. Because the normalcy and repeated measures conditions were violated for the T-test and ANOVA analysis for significance, the percent change, and Cv was calculated for both sex and age before pooling into the measurement tables. For dimensionality reduction in the longitudinal analysis, the first and second derivatives were computed for the change over time for each lesion and combined into the plotted data model. The significance of the percent change is > 5% from the initial value. The second derivative assesses for asymptotes, or steady state, where they are no further significant changes. The second derivative as an event detection model detects the trends to describe the multi-parametric longitudinal data. The CV within groups and between groups was assessed to ensure the trends were consistent within each condition.

## 3. Results

### Key findings:

**Figure 2.**
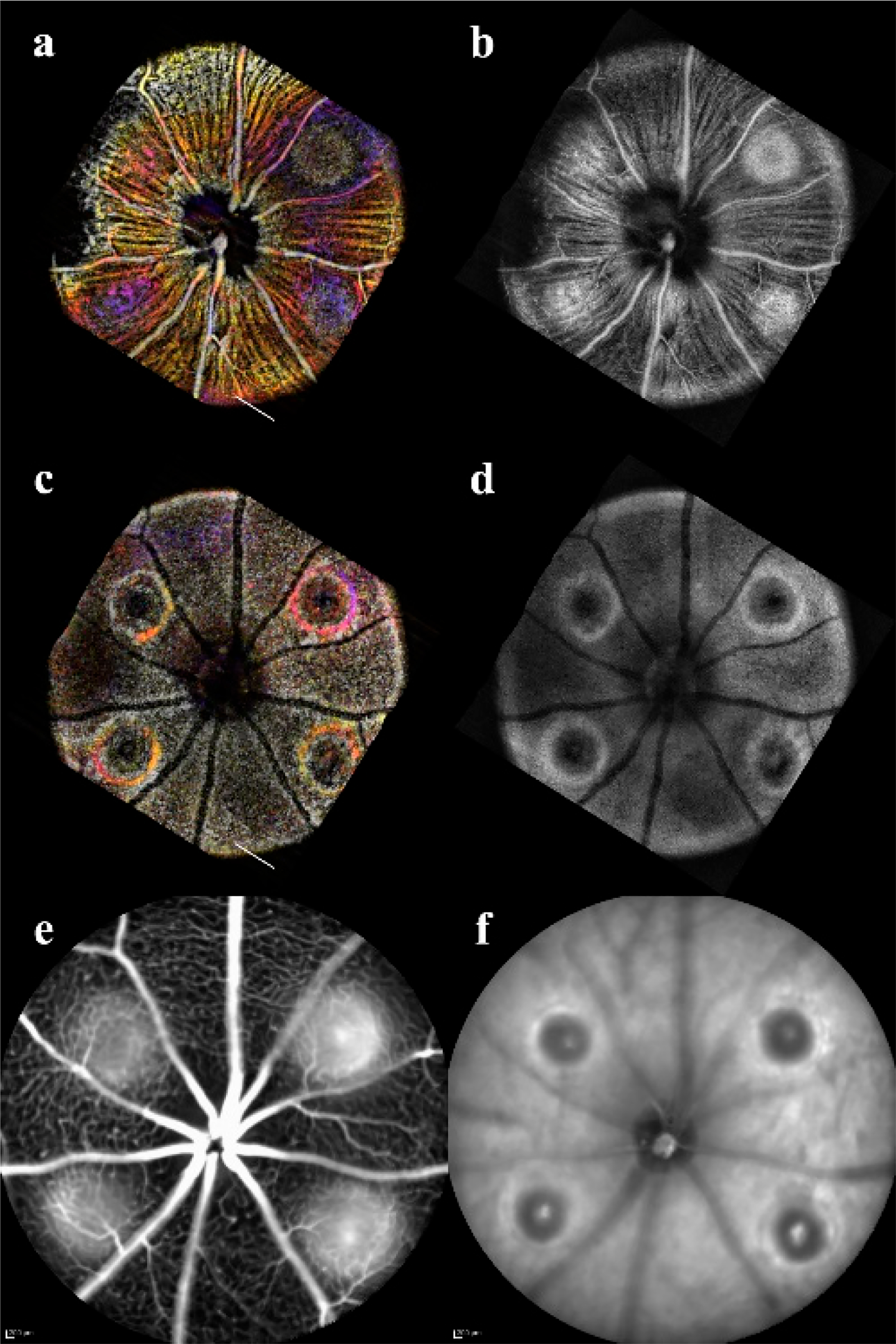
Samples of the multiple imaging views of a single eye. (**a**,**b**) is the OCTA (Optical Coherence Tomography Angiography) and en-face OCT (Optical Coherence Tomograph) of the inner limiting membrane and superficial vascular plexus. (**c**,**d**) is the OCTA and en-face view of the highlighting Retinal Pigment Epithelium and choroidal disruption. (**a**) through (**d**) are 60 μm projections of the color OCTA depth map, or maximum intensity projection of the en-face OCT. (**e**,**f**) are the fluorescein angiography and IR imaging of the retina showing the CNV (Choroidal Neovascularization) leakage. The landmarks in the en-face image (**d**) and fundus f allowed comparisons of the vasculature in the OCTA and FA images. Leakage in the CNV was correlated to the OCTA image in all cases.

**Figure 3.**
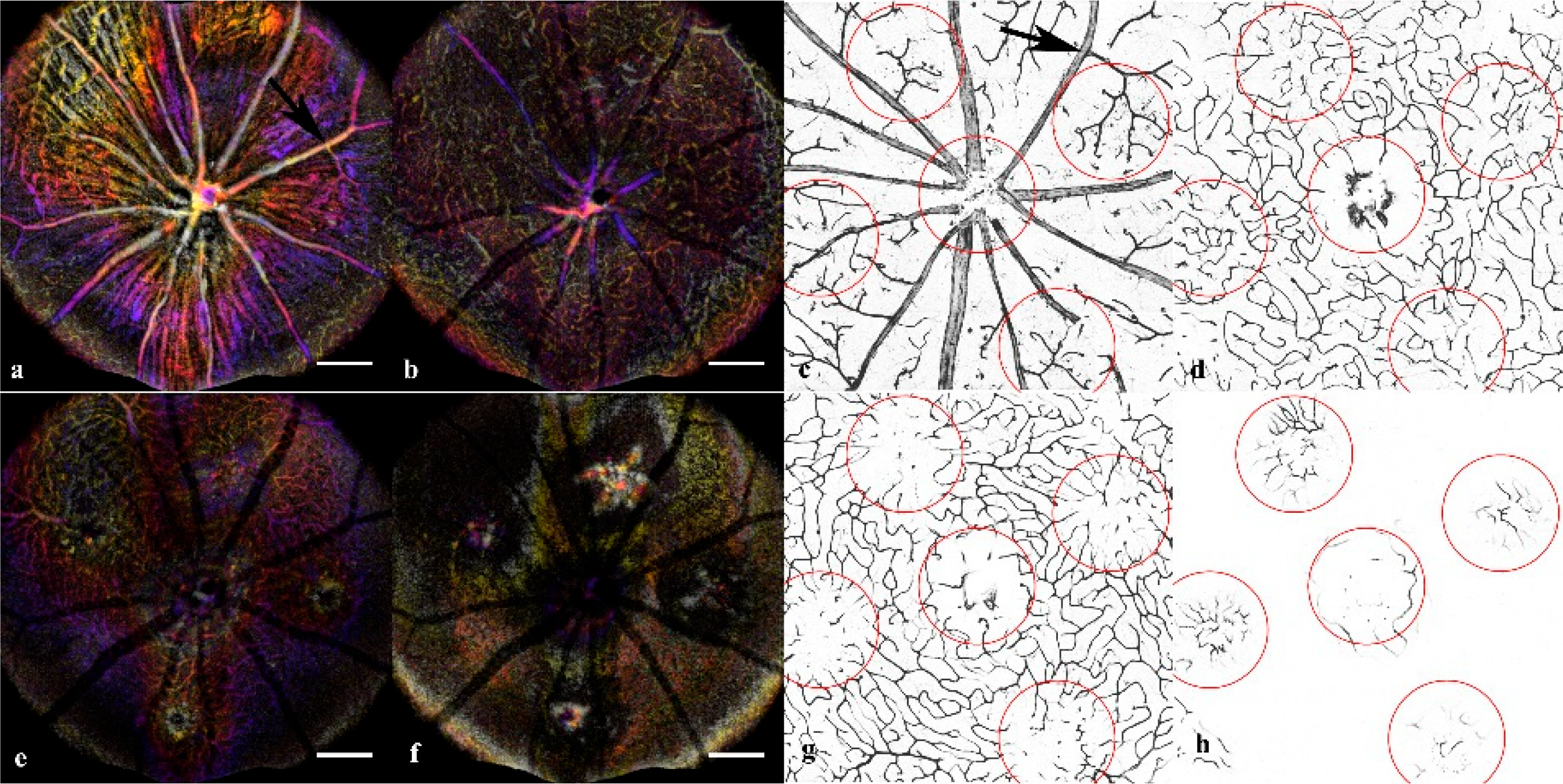
Comparisons of the in-vivo OCTA (color depth map) and retinal flatmounts (binary mask) demonstrate the correlation between findings for the SVP (Superficial Vascular Plexus), IVP (Intermediate Vascular Plexus), DVP (Deep Vascular Plexus), and ONL (Outer Nuclear Layer). At day 14 of the CNV induction, the final OCTA was generated, then perfused with Con A and extracted for flatmount. The left panel is the OCTA images represented as depth maps through the different layers. The right panel is corresponding flatmount images labeled with Con A. The Black arrow denotes a landmark vascular branch in the superficial layer (**a**,**c**) used to compare lesion locations. The superficial (**a**,**c**) and IVP (**b**,**d**) are intact with perfused vasculature at day 14. The DVP (**e**,**g**) shows a clear disruption of the vasculature where areas of non-perfusion are clear in both OCTA and confirmed in the flatmount. The ONL (**f**,**h**) should be avascular; however, at this stage of CNV, there is a signal in the OCTA and vascular staining in the flatmounts.

Orthogonal OCT allowed precise landmarking and quantification of the lesions (Figure 1) to build a mathematical model of the changes over time. The fundus screening could be replicated with en-face OCT/OCTA combination that was suited for non-invasive longitudinal studies (Figure 2). The CNV model showed not only neovascularization but loss of perfusion in the Deep Vascular Plexus that can be confirmed with immunofluorescent staining. (Figure 3).

### In-Vivo Lesion Verification:

Each lesion was classified, and measurements were compiled using the following order of assessment: fundus validation, OCT qualitative description, OCT measurements, and OCTA visualization. Following laser irradiation, all animals presented with hyperreflective lesions in the color fundus images. Acute injury dose showed no signs of bleeding or vascular leakage. The initial OCT images confirmed the presence of the lesion for both doses by the presence of a disruption in the ONL. CNV dose presented with a clear vaporization bubble in all lesions, and only one lesion had severe bleeding, examples in Figure A1 (Appendix A). The IR imaging tracked the lesions and helped guide the FA. Autofluorescence baseline imaging before FA shows an inconsistent severity of the hyperfluorescent response, some CNV lesions fluorescing brightly while others were not detectable even with maximum gain for detector sensitivity. The maximum fluorescent intensity in either model remains below the detectors threshold with the sensitivity gain used for FA. Because of the low autofluorescence intensity, it is therefore not a major confounding factor when assessing the leakage of the CNV. While the CNV had clear evidence of retinal disruptions, the boundaries of the acute injury lesions were hard to discern from the rest of the retina. Photoreceptor damage observed in the Outer Nuclear Layer (ONL) of the B-scan OCT imaging was often undetectable in the fundus images thus making us unable to determine the extent of the damage of the acute injury lesions. The orthogonal views were used to landmark the optimal B-scans for the lesions; however, any single en-face slice was insufficient to determine the morphology of the lesion.

**Figure A3.**
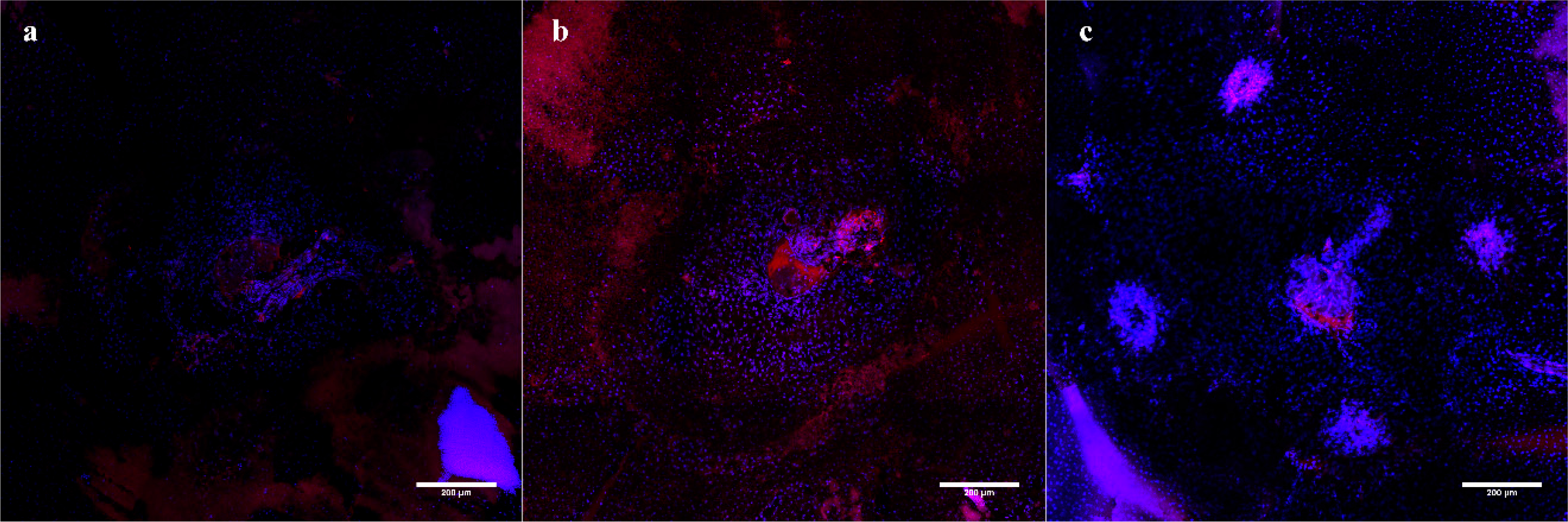
Choroid flatmounts at 1 week show the difference between the acute injury and control lesions. The acute injury choroid (**a**) is intact without visible disruptions and is comparable to the control eye (**b**). The CNV choroid (**c**) is clearly marked by four lesions and the disrupted zone around them. The DAPI (blue) and Isolectin (red) colocalize on the optic nerve head and the lesions. The control and acute injury eye have dispersed patterns of lectin and no accumulation of nuclei (note that a piece of the ONL adhered to the acute injury choroid).

From day 3, the FA scans verified the vascular leakage in the CNV lesions. The acute injury lesions showed no apparent leakage at any timepoint. In contrast, all but three of the CNV lesions clearly showed signs of leakage at all time points and could be imaged with OCT (Figure 2). The three CNV lesions that were indeterminate were noted as potentially not CNV in the continued evaluation. While three lesions did not show the characteristic pooling in FA, the normal vasculature could not be resolved either (Figure A2). All three lesions were verified as CNV with lectin in the flat mounts. The lesion that was obscured indicated bleeding and the absorption characteristics of blood severely increasing attenuation of the 488nm fluorescence; however, IR light as used in OCTA is not attenuated by blood and therefore can still resolve the lesion. Consistent with previous literature, female CNV mice developed larger initial lesions that grew proportionately to the males [34]. For longitudinal analysis 16 sample lesions for each group (CNV and acute injury) with every imaging modality imaged at all timepoints were included for all quantitative analysis. Follow-up subjects used for validation of microvascular vasculature morphometry (Figure 3) and their choroids imaged (Figure A3), and verified for consistency, but were not included in the pooled quantitative longitudinal analysis to keep group size (n = 16) consistent between time points for valid differential analysis.

### Lesion Dynamic Descriptors:

**Figure 4.**
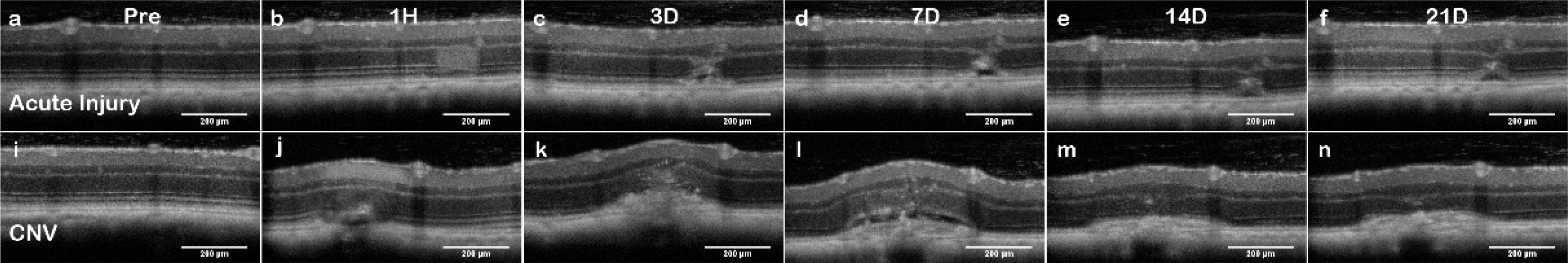
Lesion dynamics imaged by OCT over time (1 h–21 days) shows the progression of two exemplars of acute injury (**a**–**f**) and CNV lesions (**i**–**n**). The larger lesions and RPE (Retinal Pigment Epithelial) disruptions in CNV differ substantially from the subthreshold in both magnitude and temporal characteristics. Females developed more severe CNV at all time points; however, all measured trends of the pathology followed the same time course.

Orthographic views of the lesions were used to landmark and accurately extract the B-scans over time (Figure 1). Acute injury lesions exhibited a cylindrical shape, showing disruption throughout the Outer Nuclear Layer (ONL) and by day 3 began to assume an hourglass shape (Figure 4). Eventually, the hourglass neck would split, and the disruptions would form a pyramidal scar. While there was swelling, the Inner Nuclear Layer (INL) remained relatively undisturbed. It should be noted that although the RPE boundary was distorted, it remained continuous.

Early CNV lesions had less definitive borders until day 3, when the swelling was noticeable in all layers. On day 7, there was the formation of edemas with clearly defined layer separation (Figure 4). However, the few lesions that did not present with clear leakage or severe edema still had signs of angiogenesis. By day 14, a reduction in edema and scarring of RPE-ONL was more pronounced. Day 21 was marked by scarring and disruption of RPE. While all the acute injury lesions followed a predictable progression, the CNV lesions size and features were highly variable when evaluated qualitatively.

### Lesion Dynamics:

**Figure 5.**
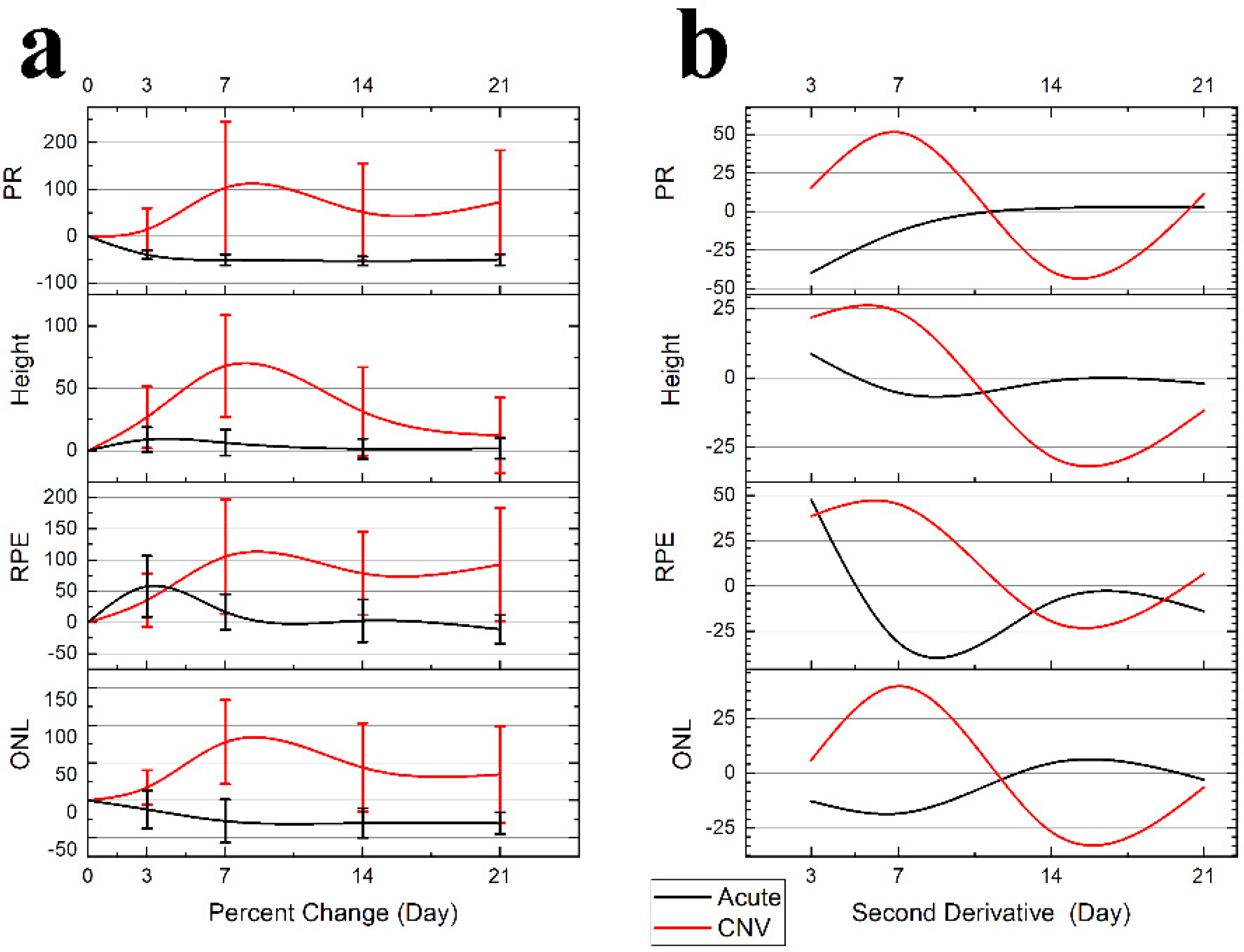
The lesion dynamics presented as first derivative percent change (**a**) and as the second derivative (**b**). (**a**): Measurements of lesion parameters (width and height) as percent change with error bars for std. The CNV (red traces) parameters all show an increase in lesion size. Parameters: ONL lateral disruption, RPE lateral disruption, height of lesion, and photoreceptor layers (PR) measuring intrusion into ONL. The Acute injury lesions (black traces) show initial swelling in the RPE layer, but peaking at day 3 and overall change to reduced lesion size reaching steady state at day 7. The height of the acute injury lesions shows no change. (**b**): The percent difference plots of the lesion measurements (ONL, RPE, height, PR) are used to assess if the lesions are reaching steady state. When the difference between two timepoints, normalized by initial measurement values approaches zero asymptote, the steady-state region. The acute injury lesions (in black) rapidly approach the zero asymptote 3–7 days. The CNV lesions (in red), are oscillating about the zero crossing at day 21.

B-Scan Measurements: The female subjects in the CNV group had more severe lesions; however, the growth trends were proportional and consistent with the males and therefore pooled for analysis presented in Figure 5 Panel A. The resulting dynamics traces, plotted in Figure 5 Panel B, were generated from pooled data of treatment groups.

The acute injury lesion is confined to the ONL with the major changes seen in the RPE distortions. (Table 1, Figure 5 Panel A) The acute injury lesion expands from 136 ± 21 μm to 196 ± 44 μm (50% change) along the RPE layer at day 3, when the trend reverses. The scar reaches steady state between day 7 and 14 with a 146 ± 45 μm lesion diameter occurring in the RPE. The PR scar is reduced from 129 ± 10 μm to 78 ± 13 μm at day 3 and reduces to a steady state at day 7. The height of the lesions shows minimal change 121 ± 10 μm to 131 ± 10 μm; therefore, the inner layers’ integrity remains continuous.

The CNV lesion show lesion dynamics with a clear transition into chronic inflammation. At day 3, all parameters are showing expansion. The lesion growth continues, and peaks around day 7. The edemas at day 7 mark the maximal vertical expansion of the lesions (Figure 4). While eventually as the swelling and edema volume subsides, only the height of the lesions returns to baseline. All other layers show clear lateral expansion of the lesion border from the first timepoint.

Table 1 lists the measurements and variance analysis for each condition. The variance measured as standard deviation was predictably higher for CNV than acute injury treatment (124 μm vs. 21 μm), so Coefficient of Variation (CV) was used to ensure consistency in comparisons. When compared, the CV of 0.19 acute injury and 0.33 CNV indicates that both sample populations were internally consistent.

When only considering inter-gender variance, the males exhibited less severe CNV and had less variance in size measurements, i.e. RPE Female 659 ± 179 μm vs. Male 397 ± 66 μm but no significant change. While the discrepancy in size increased variance of the measurement of a discrete time point, the longitudinal analysis was proportional and did not reveal any significant changes. The CV analysis results in comparable variance 0.28 ≅ 0.24; therefore, when analyzed as percent change, the trends were consistent in all lesions with no significant difference, despite variance in size (Table 1). The acute injury showed no sex discrepancy in lesion dynamics and Cv was low in all measurements. Because the lesion growth across gender is proportional to initial lesion size, the longitudinal analysis of individual lesions as percent change negates the concern of gender-induced variance in lesion size.

### En-Face and OCTA:

**Figure 6.**
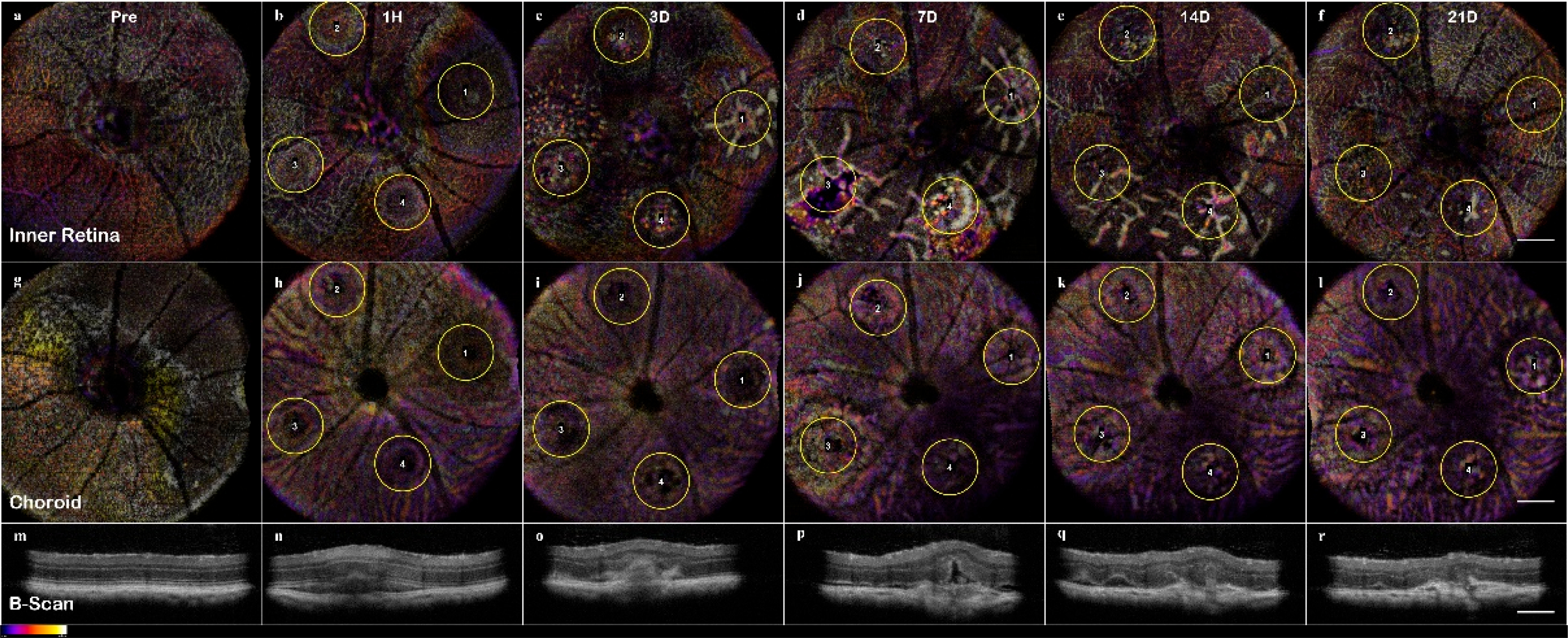
The longitudinal analysis of CNV as seen through both OCTA and High-Resolution B-Scans of lesion 1 reveals the inflammatory progression. Top row (**a**–**f**): The progression of inner retinal capillaries shows the day 3–14 non-perfusion of the vasculature as the inner layers are displaced by the edema and protrusions from the choroid. Middle Row (**g**–**l**): The choroidal layers show the lesion disruption that allows the vasculature infiltration of the retina. Bottom Row (**m**–**r**): The B-Scans of individual lesions over time tracks the swelling and edema formation in the early stage, then the neovascularization and scarring in the chronic phase. Scale bar 200 μm.

**Figure 7.**
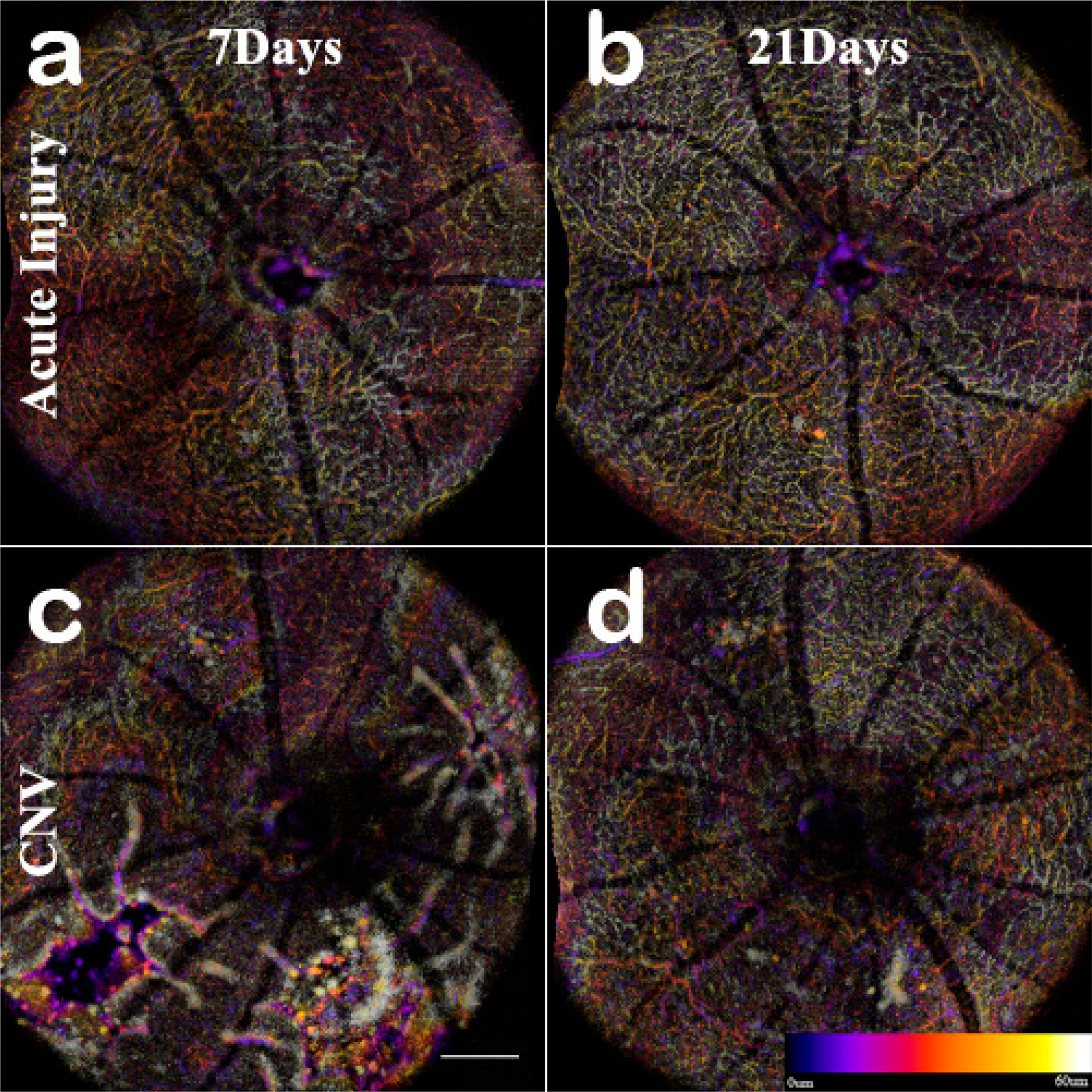
Disruptions of the inner retina capillaries are apparent when comparing the acute injury to CNV lesions. Thought the progression, the acute injury capillary bed is not disrupted (**a**,**b**). The formation of edema at day 7 in CNV (**c**) corresponds with the displacement of capillaries. At day 21 (**d**) most capillaries have returned to a normal distribution. Scale bar 200 μm.

The C-scan of the data cubes were processed with a custom algorithm [36] to spatially enhance the microvasculature. The en-face volume was assessed for morphology changes throughout the depth (Figure A2). Once features were identified, 60 μm optical slabs were projected into color depth-coded OCTA images for qualitative analysis (Figure 6). The inner retinal capillary layers identified were the Superficial Vascular Plexus (SVP), Intermediate Vascular Plexus (IVP), and Deep Vascular Plexus (DVP). The DVP (Figure 7) was most affected and analyzed in the following section (Figure A2 C). In the final images, motion artifacts still appear as banding, but the registration step minimizes the net effect. In the depth projections, the microvasculature is discernable from the background.

**Figure 8.**
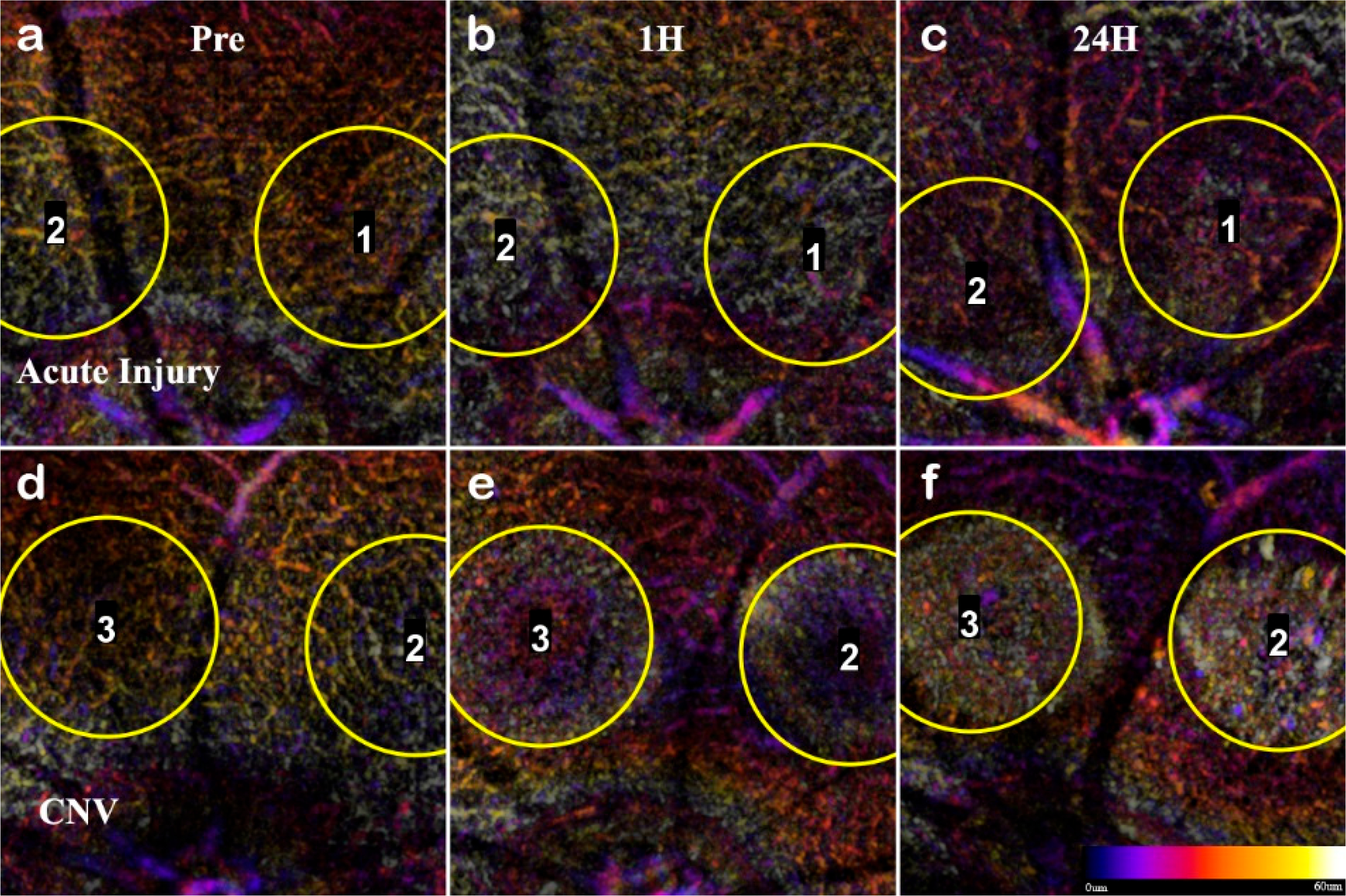
Color-coded depth-resolved projections of the SVP and DVP in the initial 24 hours following lesion induction. For each condition, the lesions were labeled in order of induction (counterclockwise) for reference. The acute injury response (**a**–**c**) does not indicate a loss of capillaries. Throughout the initial 24 hours, there was no net loss of SVP or DVP capillaries. The transient response of the inner retina vasculature in the CNV lesions (**d**–**f**) shows that non-perfusion is immediate. The CNV conditions show that the DVP capillaries are immediately disrupted at 1H (**e**), and the displacement area expands with increased swelling at 24 hours (**f**).

**Figure 9.**
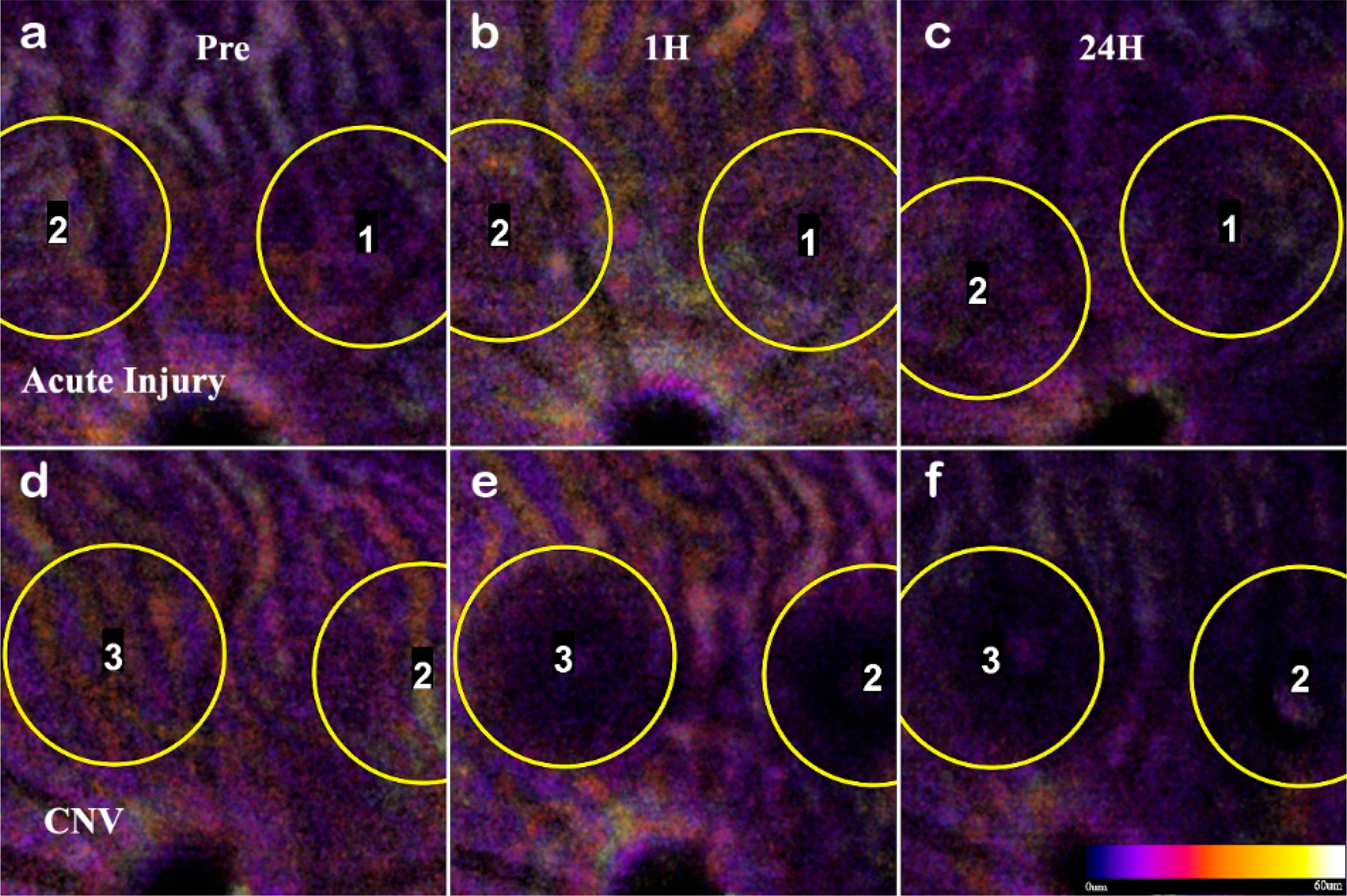
OCTA images of the Choroidal layer for acute injury lesions and CNV. While the choroid below the acute injury lesions (**a**–**c**, L1 and L2) appears to be intact, the CNV lesions (**d**–**f**, L2 and L3) clearly penetrate Bruch’s membrane appearing as a void in OCTA images. Assessment is immediate, and the separation differentiates acute insults that do not form CNV.

The acute injury lesion followed the predictable progression of an early inflammatory phase response, peaking at 3 days and reaching steady-state around day 14 (Figure 5 Panel A). The phenotype of the lesions showed initial lateral growth of the hourglass shape, before collapsing into a pyramidal scar in the ONL. The en-face OCTA data in conjunction with the B-scan data show that acute injury lesions may acutely distort the RPE layer while the Bruch’s membrane appears to remain intact. The inner retinal capillaries appear unaffected by the acute injury lesion and not an angiogenic response [47]. Moreover, for acute injury lesions, the scarring is confined to the photoreceptor layers, presenting as the lesion from the IS/OS that protrudes into the ONL yet are asymptomatic of vascular leakage when analyzed with FA. Comparing the baseline to 1-hour lesion response using the combination of B-scan and OCTA visualization, the damage of the acute injury lesions is confined to the photoreceptor layer and has not disrupted inner retinal vasculature (Figure 8) or the choroid (Figure 9). The acute injury lesions showed no gross changes in the capillary network in the OCTA scans. The acute injury lesion and scarring in the ONL slab were apparent and did not penetrate the choroid slab.

To confirm the RPE rupture and capillary non-perfusion, a repeat experimental OCTA group was set with time points 1h, 24h, and 72h. With the short time points, the choroid disruption was visible immediately and inner retina capillary non-perfusion apparent at 24h (Figure 8). The inner retina at 1 h in CNV shows displacement of layers, and at 24h the signal from the vasculature is undetectable. The acute injury samples and surrounding areas do not lose optical signal, demonstrating this is a localized disruption (Figure 9). Importantly, the integrity of the choroid slab can be immediately assessed for RPE rupture. At 1h, the acute injury lesions distort the RPE but do not penetrate through and protrude into the choroid. At 24h, the initial distortion from the localized inflammation is minimized showing choroid as a continuous layer.

In L-CNV, clear rupture of the RPE and Choroid were observed at 1 h while the retinal layers are not continuous and the damage from the vaporization bubble is apparent. The CNV lesions affected all retinal layers by displacing the inner layers and disrupting the continuity of the outer retina. The en-face view of the OCTA highlighted several key features: the rupture of the choroid, the infiltration into the ONL, and the non-perfusion of capillaries in the IPL/INL. The choroid rupture was directly correlated to the presence of leakage in the FA. None of the acute injury lesions had choroidal disruption, while the CNV lesions had disruptions proportional to the FA leakage.

**Figure 10.**
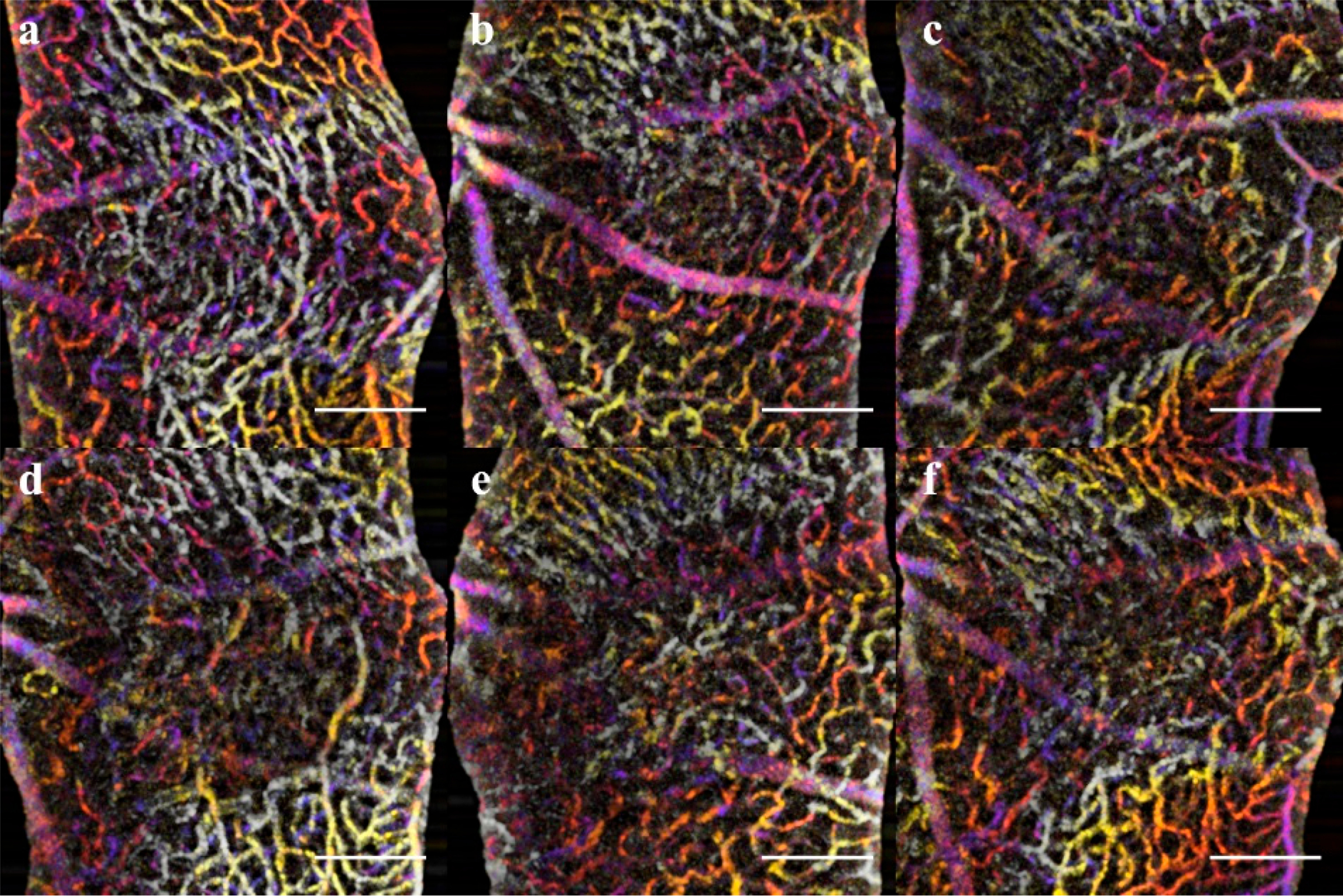
Alternative processing of the OCTA data to show the changes in the Deep Vascular Plexus over time. A single lesion was isolated in the center of the scan. (**a**) pretreatment, the entire field has clear vessels. (**b**) 1 hour, vessels in center are not well visualized. (**c**) 3 days, the vessels are disorganized or non-perfused as lesion height increases. (**d**) 7 days, vascular perfusion is low. (**e**) 14 days, perfusion can be visualized; however, the capillary organization has changed. (**f**) 21 days, a full field of capillaries returned with remodeling of the lesion.

Visualizing the CNV model through longitudinal en-face OCTA revealed that the lesions exhibited early microvascular changes before a prolonged lesion growth phase that indicates the development of chronic inflammation can be observed (Figure 10), as the lesions’ dimensions failed to reach a steady state at day 21 (Figure 5 Panel B). While most rodent CNV studies focus on days 7–21, relying on histology to confirm neovascularization [48], our results indicate there is an early dynamic process on a scale of hours to 3 days that influences both layer morphology and microvasculature. The implication is that photoreceptor layer swelling causes a deflection of the inner retina during a critical period of 1h to 3d. In this early vascular change, it is important to study how the severity of early inflammation affects the transition from acute to chronic phase 3–7days. It should be noted that the critical periods for CNV are the early acute phase 1h–3d, 3–7days for the transition to the chronic phase, and 7–21 days of the chronic phase neovascularization.

**Figure 11.**
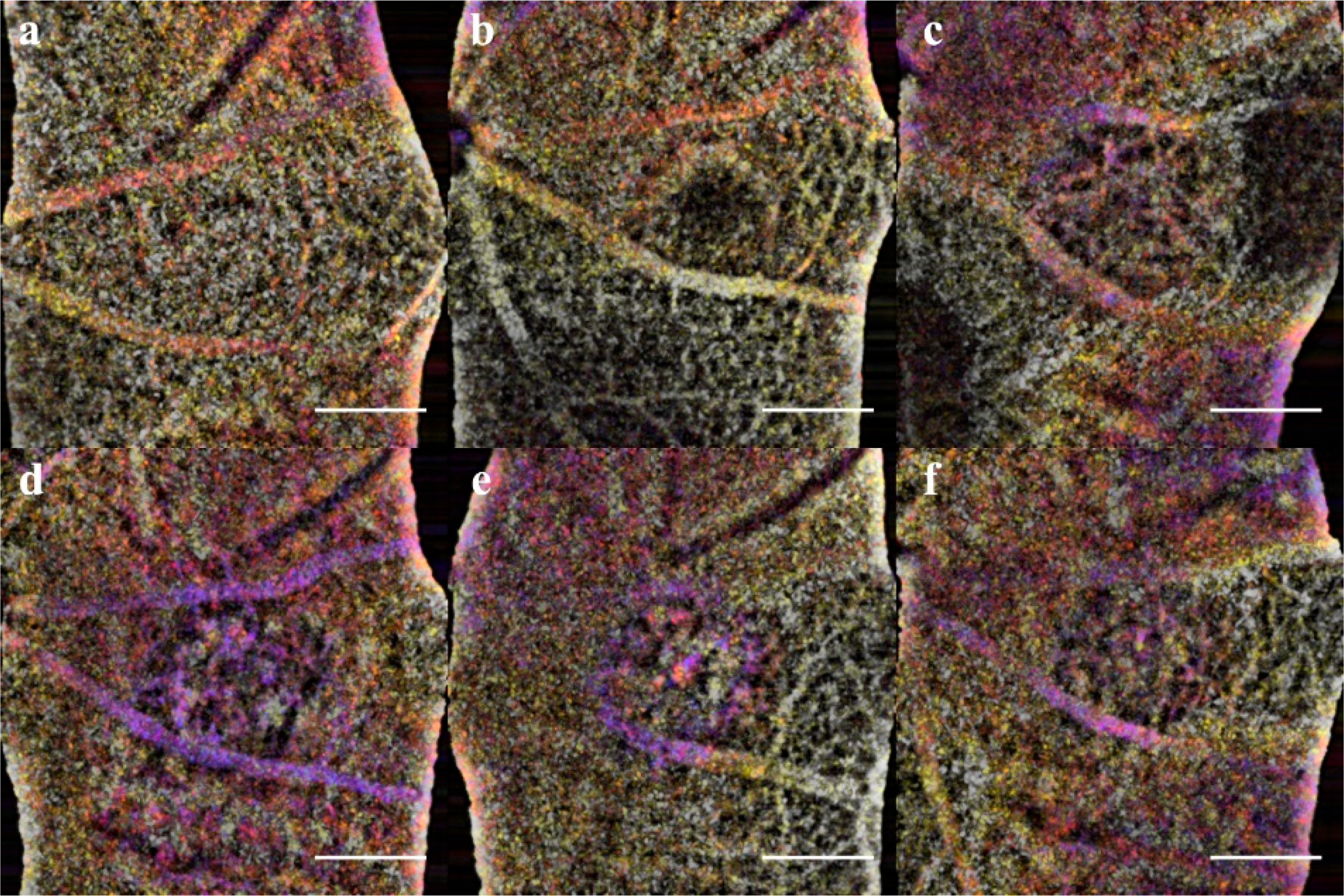
Alternative processing of the OCTA data to show the changes in the Retinal Pigment Epithelium/Choroid over time. (**a**) pretreatment shows no vasculature. (**b**) 1 hour, the initial lesion is a dark void. (**c**) 3 days, the distorted vasculature and fluid can be seen in the center of the lesion. (**d**) 7 days, A ring of neovascularization begins to form. (**e**) 14 days, The lesion forms scar and ring patterns typical of flatmounts of angiogenesis. (**f**) 21 days, the border ring is no longer visualized but infiltrating vasculature can be seen.

As evidenced by OCT assessment of the lesion, the vaporization bubble began to swell and displace the inner retinal layers immediately following the lesion induction (Figure 4). The RPE disruption was apparent at the 1h time point in the en-face OCT, corresponding with the discontinuous layers seen in the B-scan, and is an early sign of successful CNV induction (Figure 11). In non-pathogenic conditions, the acute phase response ends, and the lesion entered the resolving phase between day 3 and 7; however, in that period, the CNV development continued forming sub-retinal edemas (fully apparent at day 7) indicative of the transition into the chronic inflammatory response.

The ONL view identified the early sign of inflammation, edema formation at day 7, and eventual scarring (Figure 7). The optical slab containing the IPL/INL was most affected by the retinal swelling and edema. The region above and around the lesion was displaced, and moreover, the IVP and DVP showed regions of capillary non-perfusion. The non-perfusion was obvious on days 3 and 7; the reperfusion was not apparent until day 14 (Figures 6, 7 and 10). This capillary non-perfusion or loss of flow was not directly observable through FA, as the inner retina presents as either void or obscured by the leakage from the choroid vasculature.

### Immunofluorescent Staining:

A final group of age-matched mice was utilized for producing the retinal and choroidal flatmounts. To assess the OCTA findings in the inner retinal layers, the SVP, IVP, DVP, and ONL were mapped with OCTA projections and ConA Lectin stained flatmounts at day 14 (Figure 3). The vascular features disrupting the IVP and DVP were confirmed in the ConA Staining. Additionally, the CNV vascular intrusion into the ONL was validated in the flatmount. Neither the OCTA nor Lectin staining in the acute injury and control conditions exhibited abnormal vasculature.

Isolectin staining was used to validate the en-face OCT assessment of the integrity of the RPE layer with choroidal flatmounts (Figure A3). The flatmounts were co-stained with isolectin and DAPI to visualize the choroid. At one week, the choroid of the acute injury lesions was comparable to the naive control eyes, with neither showing signs of disruptions or abnormal vasculature. The CNV choroids showed clear neovascular scaring.

While the swelling in CNV is consistent with the previously published histopathological studies showing cellular infiltration and wound healing in the chronic phase [49], the early impact on the inner retina has not been explored. Our data shows that the early swelling and capillary non-perfusion precede edema and neovascularization. We found that the en-face OCTA provides new information on the early inner retina capillary non-perfusion in CNV (Figure 8). The severity of edema and swelling between days 3–14 correspond to the area of capillary non-perfusion that may lead to ischemia prolonging inflammation. While the choroid infiltration in CNV is expected, the inner retinal capillary non-perfusion was previously unreported.

## 4. Discussion

In retinal diseases, the vascular defects are coupled with neuronal dysfunction. The ischemic environment of the retina is easily perturbed by an inflammatory insult. Loss of vasculature or increased ischemia can dysregulate the normal anti-angiogenic signaling and instead, promote neovascularization [42,50]. The laser lesions used in this study formed either an acute injury or chronic response contrasted by the progressive inflammation injury that induces choroidal neovascularization. The acute injury is a predictable model for the early pro-inflammatory phase of lesion formation and progression [14,51], as supported by the low variance in the lesion sizes (measured by Cv in Table 1) in contrast to the uncontrolled angiogenesis characteristic of CNV. Determining the features of the inflammation progression and alterations to the vascularization are of important to understanding neuroinflammation specifically, when changes might occur that transform an injury into neurodegeneration.

In CNV model, the physical damage and retinal infiltration vasculature displacing the photoreceptors are well established [38,52], but the progression of the vascular inflammatory response and inner retinal neurons is unknown. Traditional angiography methods cannot assess how laser injury in the two models affects the remodeling of inner retinal capillaries or implications to the degree of ischemia in the eyes. The en-face OCT scans provide a non-invasive approach to assess how outer retinal swelling associated with an inflammatory stimulus affects the inner retina. This longitudinal study of an acute and chronic injury reveals how the progression of layer disruption corresponds to vascular changes.

With the non-invasive nature of OCT, we successfully demonstrated the ability to track individual lesions in a longitudinal study designed to identify key timepoints in the progression of lesion morphology that differentiates an acute injury lesion from CNV pathology (Figure 4). Through the high resolution, B-scan images were generated from the registered volume (Figure 1) enabling optical sectioning of the center of each of the lesions so that for each timepoint repeatable lesion dimensions were reliably calculated [23,53]. From these measurements, we determined that the laser irradiation parameters (~0.01 Joules) induced not only increased lesion size but changes in both pathology and duration of inflammatory response. Plotting the derivative, the percent difference between timepoints for each of the lesion measurement data (Figure 5), the peak response and transition into the resolving phase begins by day 3 in the acute injury model, although the CNV response continues as both inner and outer vascular networks are remodeling in the chronic phase. The features seen in the B-scan images indicate that the CNV lesions all have swelling that displaces the inner retina, and edema is formed at day 7. In addition, this method which allows for digital re-sectioning yielded simultaneous lesion measurements and OCTA visualization. The combination provided improved capability for assessing the impact of swelling upon layer morphology and the subsequent disruptions to the capillary network.

While the chronic inflammation and leaky vasculature are well-established features of wet AMD, the inner retinal changes are not well characterized. At 21 days, the OCTA images of the choroid were consistent with the leakage area in FA (Figure A1); however, in the early response at 3–14 days, the OCTA of the inner retina exhibited capillary non-perfusion and reperfusion that directly correlates with the swelling and edema in CNV when the lesion height ≥200 μm. The finding presented in a OCTA survey of macular edema due to retinal branch vein occlusion, supported correlation but not indicative of causality between edema and vascular rarefaction [54]. With the loss of continuous flow to generate contrast in the en-face OCT, the vasculature signal becomes fragmented or completely lost. Although inner capillary dropout is a known contributing factor to retinal ischemia in glaucoma and diabetic retinopathy visible through OCTA [55], it has not previously been attributed as a feature of CNV or AMD. With the swelling and edema in the early phases of CNV, the displacement of the inner layers may cause the vasculature to be pinched, restricting or stopping blood flow altogether.

The CNV lesion dynamics of the RPE and PR layers in the chronic phase correlate to the in-vivo findings of previous studies [27,56], indicating that the resulting pathology exhibits similar features in all studies. Therefore, the combined orthogonal OCT and OCTA imaging approach allow for the study of how the swelling and edema in inflammation progression causes displacement that affects the microvasculature of the inner retina. Furthermore, the method of post-processing allows for comparison of the dynamics of acute vs. chronic injury and specifically offers a longitudinal method to non-invasively correlate the edema formation to vascular changes.

The contrast of the vasculature visualized with OCTA is dependent on the spatial/temporal speckle pattern of erythrocytes in blood flow. The algorithm we developed utilizes the spatial domain component of OCT speckle patterns to highlight and extract the angiogram from an en-face OCT volume [36,57]. The high-density volumetric scanning of en-face OCT allows for digital re-slicing and processing with a custom 3D vessel enhancement algorithm extending the principles of vessel detection strategies for fundus images [58]. Our OCTA methodology allowed the findings in this study to be confirmed across subjects by re-slicing the volumes to focus on the inner retina vasculature that was perfectly registered to the high-resolution B-scans of the lesions. Additionally, by using a single high-density scan and the spatial portion of the speckle pattern, instead of repeated scans, the algorithm covers a wider area. The tradeoff to the approach is that the flow rate cannot be calculated through this method. However, currently flow rate cannot be validated, thereby large area screening of vascular morphology was a better utilization of the data. As a variant of the speckle decorrelation algorithm, our en-face OCTA methodology is capable of resolving the microvascular morphology comparable to that which was observed in the previous rodent studies [32].

In human CNV studies using OCTA, only the choriocapillaris was compared to the FA imaging [27, 59], and OCTA is highly correlated to the leakage. While FA is used clinically to determine the presence or absence of vascular leakage or edema, lectin staining is used in pre-clinical trials for measuring vascular dysfunction indirectly as leakage area. Lectin staining on rat retinal flatmount has shown that there is no increase in lesion growth after day 10; however, such methods cannot assess the functional aspect of microvasculature perfusion under in-vivo conditions [37]. Our longitudinal analysis of the inner retina non-perfusion and reperfusion was only possible using an OCTA methodology. OCTA with blood flow velocity analysis or new oximetry techniques [60,61] is required to verify that there is a temporary loss of the blood perfusion increasing localized hypoxia.

## 5. Conclusions and Perspectives

In this study, we demonstrated the potential utilities of combined en-face OCTA and the high-resolution B-Scans for non-invasive longitudinal assessment of progression of laser-induced injury in the retina in the small animal model. The orthographic analysis of lesion and OCTA detects features that are indicative of phases of the inflammatory response of retina. We utilized differential analysis of the lesion measurements to quantify the changes over time, detecting the phases with differential analysis. The image-based features offer desirable capability needed to investigate the transition from the early transient swelling (≥ 200 μm) with a previously unidentified capillary non-perfusion (localized in the IVP/DVP microvasculature), to the later edema formation that leads to scaring and CNV. The acute injury model demonstrated that the photoreceptor ablation occurred without inducing microvascular changes. The vascular non-perfusion and remodeling in the CNV model is a new finding identified in the L-CNV injury. The non-perfusion is not a direct impact of laser damage disrupting the inner retinal vasculature as it is not present in the acute injury lesions with an equivalent total energy (fluence = intensity × time). Furthermore, the orthographic/OCTA methodology was able to confirm that in the acute model, the microvasculature was not disrupted, and the injury was predominantly photoreceptor damage. Therefore, the depth-resolved visualization of the vascular network with OCTA is a label-free imaging technique allowing for developing an improved understanding of the vascular layer involvement retinal pathology.

Vascular remodeling is a crucial component of inflammation which could not be both non-invasively and volumetrically assessed using conventional angiography techniques. However, orthographic/OCTA can track pathology and resolve the capillary alterations over time, further enabling the identification of key phases of the inflammatory progression. The two models separated the acute inflammatory response and the chronic response, highlighting days 3–7 as a critical transition phase. While this study focused on the assessment and monitoring of laser-induced damage, the methodology applies to many other retinal diseases with angiogenic components. The assessment of subtle microvascular remodeling is important for monitoring retina health due to the high oxidative demand and avascular inner layers, such as those exhibited in diabetic retinopathy and glaucoma.

## Figures and Tables

**Table 1.** Table of average lesions dimensions, measurement in μm, as the lesions change over time. The Coefficient of Variation (CV) is standard deviation normalized by the mean, σ/μ, is used to compare the measurement consistency. The Acute injury measurements show that the lesions are substantially smaller at day 1, 128 μm compared to 408 μm. At 21 days, acute injury measurements show a decrease while CNV has expanded.

			Acute injury					

**Day**	**ONL**	**CV**	**RPE**	**CV**	**Height**	**CV**	**PR**	**CV**

1	128.31	0.10	136.59	0.16	121.39	0.06	129.71	0.08
3	110.90	0.22	196.09	0.23	131.55	0.08	78.07	0.18
7	87.86	0.36	156.98	0.19	125.08	0.10	60.96	0.20
14	94.38	0.32	146.75	0.31	123.95	0.11	64.52	0.25
21	94.50	0.28	137.17	0.33	121.55	0.08	69.13	0.24

			CNV					

**Day**	**ONL**	**CV**	**RPE**	**CV**	**Height**	**CV**	**PR**	**CV**

1	408.75	0.24	345.66	0.50	170.38	0.19	276.72	0.43
3	418.96	0.24	428.85	0.13	204.83	0.19	278.93	0.25
7	566.08	0.34	566.49	0.32	246.35	0.32	401.73	0.50
14	457.69	0.39	520.06	0.30	197.74	0.29	314.71	0.49
21	433.87	0.43	528.33	0.37	178.06	0.26	342.27	0.52
